# Exploiting a living biobank to delineate mechanisms underlying disease-specific chromosome instability

**DOI:** 10.1007/s10577-023-09731-x

**Published:** 2023-08-17

**Authors:** Louisa Nelson, Bethany M. Barnes, Anthony Tighe, Samantha Littler, Camilla Coulson-Gilmer, Anya Golder, Sudha Desai, Robert D. Morgan, Joanne C. McGrail, Stephen S. Taylor

**Affiliations:** 1 Division of Cancer Sciences, School of Medical Sciences, Faculty of Biology, Medicine and Health, University of Manchester, Manchester Cancer Research Centre, Wilmslow Road, Manchester, M20 4GJ UK; 2 Department of Histopathology, The Christie NHS Foundation Trust, Wilmslow Rd, Manchester, M20 4BX UK; 3 Department of Medical Oncology, The Christie NHS Foundation Trust, Wilmslow Road, Manchester, M20 4BX UK

**Keywords:** Chromosome instability, tumour heterogeneity, cancer genomics, ovarian cancer

## Abstract

Chromosome instability (CIN) is a cancer hallmark that drives tumour heterogeneity, phenotypic adaptation, drug resistance and poor prognosis. High-grade serous ovarian cancer (HGSOC), one of the most chromosomally unstable tumour types, has a 5-year survival rate of only ~30% — largely due to late diagnosis and rapid development of drug resistance, e.g., via CIN-driven *ABCB1* translocations. However, CIN is also a cell cycle vulnerability that can be exploited to specifically target tumour cells, illustrated by the success of PARP inhibitors to target homologous recombination deficiency (HRD). However, a lack of appropriate models with ongoing CIN has been a barrier to fully exploiting disease-specific CIN mechanisms. This barrier is now being overcome with the development of patient-derived cell cultures and organoids. In this review, we describe our progress building a *Living Biobank* of over 120 patient-derived ovarian cancer models (OCMs), predominantly from HGSOC. OCMs are highly purified tumour fractions with extensive proliferative potential that can be analysed at early passage. OCMs have diverse karyotypes, display intra- and inter-patient heterogeneity and mitotic abnormality rates far higher than established cell lines. OCMs encompass a broad-spectrum of HGSOC hallmarks, including a range of p53 alterations and *BRCA1/2* mutations, and display drug resistance mechanisms seen in the clinic, e.g., *ABCB1* translocations and *BRCA2* reversion. OCMs are amenable to functional analysis, drug-sensitivity profiling, and multi-omics, including single-cell next-generation sequencing, and thus represent a platform for delineating HGSOC-specific CIN mechanisms. In turn, our vision is that this understanding will inform the design of new therapeutic strategies.

## Introduction

Many human tumours are characterised by extensive copy number variation (CNV), which arises due to an underlying chromosome instability (CIN) phenotype (Ciriello et al. 2013). CIN leads to continuous gain and loss of chromosomes and/or acquisition of structural rearrangements, in turn driving tumour heterogeneity, phenotypic adaptation and drug resistance (Patch et al. 2015; Schwarz et al. 2015; McPherson et al. 2016; Naffar-Abu Amara et al. 2020; Vasudevan et al. 2020; Ippolito et al. 2021; Lukow et al. 2021). Despite an intense focus on the causes of CIN, we still do not understand the full spectrum of molecular drivers, possibly reflecting the presence of multiple mechanisms and/or disease-specific CIN drivers.

**Fig. 1:**
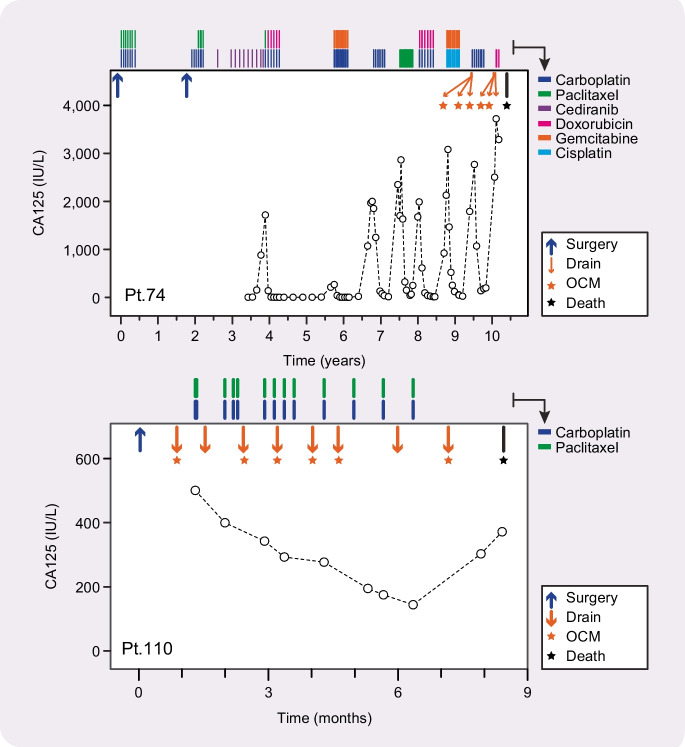
Treatment timelines of patients with HGSOC. Line graphs showing CA125 levels, measured via blood sampling, for patients 74 and 110 over time following diagnosis. Graphs are annotated to show surgery (blue up arrows), when ascites were collected (orange down arrows), which ascites generated OCMs (orange stars) and when the patient died (black star). Vertical coloured bars along the top of the plot area show the timing of the indicated chemotherapy treatments

Our focus is on high-grade serous ovarian cancer (HGSOC); one of the most chromosomally unstable cancer types (Ciriello et al. 2013). HGSOC is the commonest histological subtype of ovarian cancer, representing approximately 80% of all cases (Jayson et al. 2014). It is frequently diagnosed at an advanced stage having already undergone metastatic spread beyond the pelvic intraperitoneal tissues. While most cases initially respond to chemotherapy, most women will develop drug-resistant disease (Clamp et al. 2019) (Fig. 1). A known driver of CIN is defective DNA damage repair, and in the case of HGSOC, possibly up to 50% are homologous recombination deficient (HRD), frequently caused by mutations in the *BRCA1* and *BRCA2* tumour suppressor genes (TCGA 2011; Denkert et al. 2022; Morgan et al. 2023a). Almost 20 years ago, a major advance was the discovery that *BRCA1/2*-mutant cells are exquisitely sensitive to PARP-1/2 inhibitors (PARPi) (Bryant et al. 2005; Farmer et al. 2005), paving the way for new therapeutic strategies that have had a major beneficial impact in the clinic (Mirza et al. 2016; Coleman et al. 2017; Pujade-Lauraine et al. 2017; Moore et al. 2018; Gonzalez-Martin et al. 2019; Monk et al. 2022). As such, PARPi provide an excellent paradigm illustrating how CIN mechanisms can be exploited to improve patient outcomes. Further exploitation of HGSOC CIN will be important because, in addition to the paucity of actionable oncogenic mutations, at most only 50% are HRD and thus predicted to respond to PARPi. A major research goal therefore is to define the spectrum of CIN mechanisms in HGSOC to identify additional tumour-cell-specific vulnerabilities that can be therapeutically exploited.

Delineating the full spectrum of CIN mechanisms will require model systems that reflect the diverse CIN phenotypes observed in human tumours. Studies on established human cancer cell lines have been instrumental in dissecting some aspects of CIN mechanisms, and importantly CIN manifests in HGSOC-derived cell lines, with evidence of both mitotic defects and DNA replication stress (Penner-Goeke et al. 2017; Nelson et al. 2020; Tamura et al. 2020). However, while cell lines are experimentally tractable, they have several weaknesses. Often, cell lines were established decades ago in sub-optimal culture conditions that may have selected specific phenotypes (Domcke et al. 2013; Ince et al. 2015; Nelson et al. 2020). Further propagation in vitro likely selects out the fitter subclones best adapted to cell culture conditions, possibly eliminating lesser fit clones that might only survive in vivo. Established cell lines also often lack detailed clinical annotations (e.g., histology, chemotherapy exposure and/or clinical response), and matched pre- and post-treatment lines are rare.

**Fig. 2:**
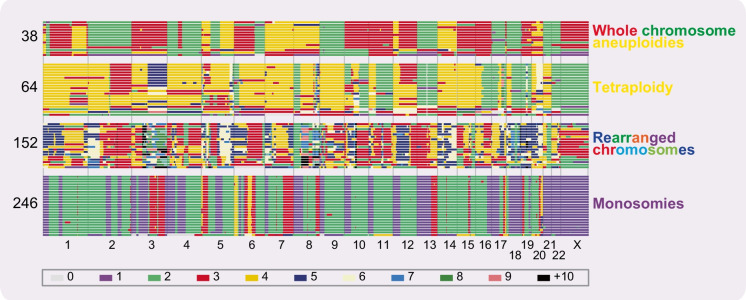
HGSOC is characterised by extensive chromosome instability. Genome-wide chromosome copy number profiles determined by shallow single-cell whole-genome sequencing (scWGS) of OCMs derived from patients 38, 64, 152 and 246. For each OCM, each row represents a single cell, with chromosomes plotted as columns and the copy number indicated by the colour. The four OCMs shown represent examples whereby genomes are marked by whole-chromosome aneuploidies, rearranged chromosomes, tetrasomies or monosomies. Karyotypes previously shown in Nelson et al. 2020 and Coulson-Gilmer et al. 2021 (Licenses at https://creativecommons.org/licenses/by/4.0/)

A major advance addressing some of the limitations of established cell lines is the development of *Living Biobanks*, collections of patient-derived cell cultures or organoid models that are clinically annotated and better capture the heterogeneity observed in human tumours. A key development came from colorectal cancer (CRC), with the discovery of culture techniques that allowed expansion of CRC tumour cells in organoid structures (Sato et al. 2011). This technology has now been extended to other cancers (Gao et al. 2014; Boj et al. 2015; Sachs et al. 2018), including ovarian cancer (Kopper et al. 2019; de Witte et al. 2020). In this review article, we describe our experience developing a *Living Biobank* of patient-derived ovarian cancer models (OCMs) (Nelson et al. 2020). OCMs are highly purified tumour fractions that have extensive proliferative potential and can be analysed at early passage. They have highly diverse karyotypes, displaying extensive intra- and inter-patient heterogeneity that falls into several subclasses (Fig. 2), and as such provide an attractive starting point for delineating CIN mechanisms.

## Solid sampling versus ascites collection

**Fig. 3:**
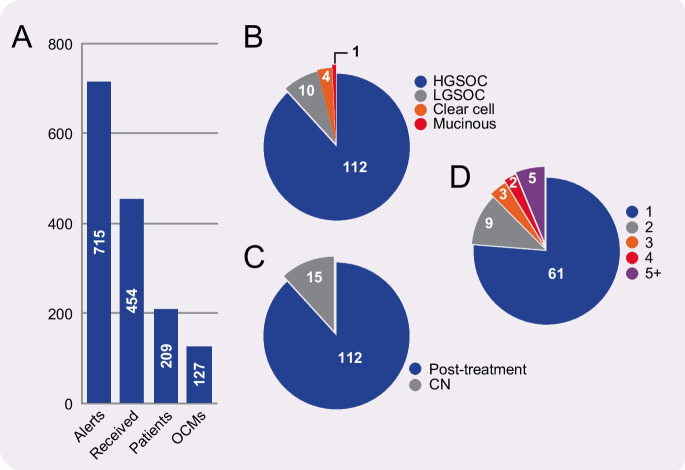
Living Biobank ascites pipeline. **A** Bar graph summarising the biopsy pipeline, showing that from June 2016 to March 2023, 715 biobank alerts yielded 454 ascites samples from 209 patients, in turn yielding 127 OCMs. **B–D** Summary of OCM collection with pie charts showing breakdown of subtypes based on pathology assessment (**B**); chemo-naïve (CN) vs. post-treatment (**C**); and longitudinal subsets (**D**). In (**D**), while 61 patients are represented by a single OCM (blue), 9 patients have 2 longitudinal OCMs (grey), and 3 patient subsets have 3 longitudinal OCMs (orange), etc.

Standard treatment for ovarian cancer is cytoreductive surgery followed by platinum-based chemotherapy (Jayson et al. 2014), with ~60% of patients in the UK receiving neo-adjuvant chemotherapy. Beyond that, maintenance therapy includes the PARPi olaparib or niraparib, the anti-angiogenic agent bevacizumab, or olaparib plus bevacizumab. Following relapse, a variety of chemotherapeutic strategies can be used, with treatment decision often based on the platinum-free interval (McGee et al. 2017) (Fig. 1). Secondary cytoreductive surgery is less common, but ascites will frequently be removed for symptomatic benefit using therapeutic abdominal paracentesis. Our biopsy pipeline delivers both solid surgical samples and ascitic fluid, and we have developed OCMs from both (Nelson et al. 2020), albeit with a clear bias towards ascites (Fig. 3).

For histological diagnosis and molecular characterisation, solid specimens (tumour tissue either from a diagnostic biopsy or surgical resection) are often considered the gold standard. Key advantages are the ability to sample both primary and metastatic sites, and — via analysis of spatially resolved tumour material — probe heterogeneity between sites and enable the reconstruction of evolutionary trajectories (Schwarz et al. 2015; McPherson et al. 2016; de Witte et al. 2020; Burdett et al. 2023). Because secondary cytoreductive surgery occurs infrequently in the treatment of HGSOC, solid biopsies have limited potential to deliver temporally resolved samples. Furthermore, single surgical samples may not fully capture disease heterogeneity (Hoogstraat et al. 2014; Schwarz et al. 2015; McPherson et al. 2016; Morgan et al. 2023b). In terms of isolating viable tumour cells, solid biopsies can be challenging when tumour material is limited, e.g., with core diagnostic biopsies, and especially following neo-adjuvant chemotherapy, where 60–70% of patients achieve a response, i.e. little or no primary tumour remaining (Morgan et al. 2021). At this point, specimens can be non-viable and so generating a culture is unlikely. However, if grossly visible tumour material is present and/or tumour-rich regions can be isolated by microdissection, ex vivo cultures can be developed even following neoadjuvant chemotherapy (Hill et al. 2018). Another limitation of surgical biospecimens is that they may come from early-stage disease that is typically cured with surgery plus platinum chemotherapy (Trimbos et al. 2003; Collinson et al. 2014). However, death from advanced HGSOC is commonly associated with chemotherapy-resistant disease, which emerges many months or even years later and is not captured at the time of primary cytoreductive surgery (Fig. 1).

The accumulation of ascites presents an alternative method to sample ovarian cancer cells. The presence of tumour cells in the peritoneal cavity can drive fluid build-up by VEGF-mediated increase in capillary permeability and compromised lymphatic drainage (Kipps et al. 2013; Ford et al. 2020). In turn, cytokines, chemokines, and growth factors present in ascites can promote tumour cell survival and further metastatic spread. Excessive fluid is frequently drained for symptom control and, because ascites contains large numbers of tumour cells, it provides excellent opportunities for translational research. Moreover, because abdominal paracentesis provides a safe method for repeat sampling, it opens the opportunity to collect longitudinal samples, including chemo-naïve and spanning multiple treatments (Fig. 1). Because HGSOC is often diagnosed late, when up to 90% of patients will develop ascites (Huang et al. 2013; Ford et al. 2020), this method can capture a wide spectrum of disease. Ascitic fluid can also capture intra-tumour heterogeneity, with one study demonstrating that >92% of somatic mutations detected across multiple intra- and extraovarian solid lesions were represented in ascites-derived tumour samples (Choi et al. 2017). Moreover, genomics datasets from primary disease (generally solid) and chemo-resistant disease (generally ascitic) are largely consistent (TCGA 2011; Patch et al. 2015). In terms of probing biology and exploring therapeutic strategies, ascites collection permits access to chemotherapy-resistant disease, since resistant tumour cells may be absent or represent only a minor proportion of primary cytoreductive surgical samples.

## Optimisation of culture media

The development of better experimental models to study ovarian cancer is a major research focus (Bowtell et al. 2015). Indeed, as reviewed recently (Tomas and Shepherd 2023), extensive effort has been applied to develop patient-derived 2D cell cultures, more complex spheroid, organoid or co-culture models, as well as xenografts (Bertozzi et al. 2006; Shepherd et al. 2006; Latifi et al. 2012; Sueblinvong et al. 2012; Thériault et al. 2013; Davidowitz et al. 2014; Lengyel et al. 2014; Ince et al. 2015; Liu et al. 2017; Thu et al. 2017; Hill et al. 2018; Kopper et al. 2019; Maru et al. 2019; Phan et al. 2019; Fritz et al. 2020; Hoffmann et al. 2020; Maenhoudt et al. 2020; Brodeur et al. 2021; Ito et al. 2023; Vias et al. 2023). Such efforts have been required because establishing primary cell cultures from tumours using traditional cell culture techniques has historically been challenging, with very low success rates due to tumour cell ‘senescence’ and with the emerging cell lines reflecting rare subclones (Ince et al. 2015). A major breakthrough was the development of highly specialised cell culture conditions capable of propagating isolated CRC cells as organoids (Sato et al. 2011), an approach then adapted to breast (Sachs et al. 2018) and epithelial ovarian cancers (Kopper et al. 2019; de Witte et al. 2020). A parallel breakthrough was the development of Ovarian Carcinoma Modified Ince (OCMI) media, by T Ince, with J Brugge, G Mills and colleagues, which allows propagation of epithelial ovarian cancer cells as 2D monolayers (Ince et al. 2015). Prior to adopting OCMI, our attempts to develop proliferative ex vivo HGSOC cultures were unsuccessful; while fibroblasts isolated from ascites proliferated in traditional RPMI-based formulations, the associated tumour cells did not. Adopting OCMI had a transformative effect; as of March 1st, 2023, we have received 454 ascites samples from 209 patients and thus far generated 127 OCMs (Fig. 3). The ‘take-rate’ at first pass is approximately 30% and, in some cases, OCMs have been generated following second and third attempts by fine-tuning initial conditions. Importantly, the vast majority of OCMs can also be revived after cryopreservation; thus far only two OCMs do not revive. Generation of highly purified tumour fractions is usually possible in under five passages, allowing extensive analyses on early passages. If the tumour cells are p53-deficient, this process can be accelerated by selectively killing p53-proficient stromal cells with Nutlin-3 (Nelson et al. 2020). OCMs cultured in OCMI have extensive proliferative potential, with some propagated beyond 50 passages. Prolonged propagation is anticipated to select for the fitter, faster growing subclones that may be more chromosomally stable over time (Nelson et al. 2020). Indeed, in due course OCMs are anticipated to behave like established cell lines.

## Ex vivo cultures retain the hallmark characteristics of HGSOC

A key question is whether ascites-derived OCMs reflect the primary tumour. While at first this question seems straightforward, upon closer inspection it is more nuanced. In many cases, OCMs and the corresponding primary tumour are separated by many months if not years (Fig. 1). Considering both the extensive genomic plasticity caused by CIN, and significant selection pressures exerted by multiple rounds of chemotherapy, one might expect the tumour cells sampled in ascites to have diverged considerably from the original primary tumour.

**Fig. 4:**
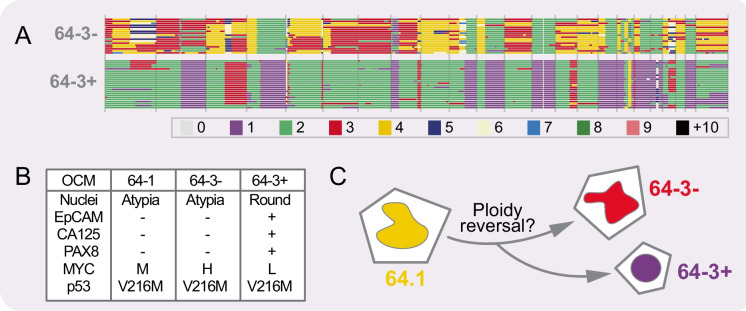
Chromosome instability generates highly divergent subclones. **A** scWGS-derived karyotypes of EpCAM-positive and EpCAM-negative subpopulations present in the OCM generated from the 3^rd^ ascites sample collected form patient 64. **B** Table summarising characteristics of OCMs 64-1 and the two 64-3 subpopulations. **C** Speculative ploidy reversal event to explain how the two 64-3 subpopulations might have been generated. Karyotypes in **A** adapted from Nelson et al. 2020 (License at https://creativecommons.org/licenses/by/4.0/)

OCMs derived from patient 64 illustrate this incredible plasticity (Nelson et al. 2020). OCMs 64-1 and 64-3 were generated from ascites collected from the same patient 49 days apart, the first and third abdominal drains respectively, almost 2.5 years after surgery. Microscopy revealed that many cells in OCM.64-3 had similar morphology to those in OCM.64-1, with large, atypical nuclei, and negative PAX8 and EpCAM expression (Fig. 4). However, we also identified a second population in OCM.64-3 that had smaller nuclei and were positive for both PAX8 and EpCAM. By exploiting the differential EpCAM status, we physically separated the two sub-populations to create OCM.64-3-Ep+ and OCM.64-3-Ep-. This revealed that the EpCAM-negative population expressed high levels of MYC and had a gene expression profile that more closely resembled OCM.64-1 (Fig. 4).

Karyotype analysis revealed that OCM.64-1 was dominated by tetrasomies (Fig. 2) (Nelson et al. 2020). By contrast, OCM.64-3-Ep- harboured disomies and trisomies, while OCM.64-3-Ep+ harboured numerous monosomies (Fig. 4). Importantly, the p53 mutation — p.V216M — was identical, and unique in the collection to date, indicating a clonal origin (Fig. 4). Interestingly, the disomies in OCM.64-3-Ep- were mirrored by monosomies in OCM.64-3-Ep+ (Fig. 4). One possible explanation is that an unequal mitosis resulted in a ploidy reversal event, giving rise to the two cell types found in 64-3 (Fig. 4). Note that ploidy reversal has been described in polyploid hepatocytes as part of a mechanism to generate genomic diversity (Duncan 2013). If ploidy reversal did occur, this would represent an additional mode of punctuated tumour cell evolution, yielding very rapid genomic divergence. Nevertheless, this subset of OCMs illustrates the remarkable plasticity of HGSOC cells in terms of key tumour markers, gene expression profiles and karyotype. In turn, illustrating that perhaps beyond truncal *TP53* mutations, we should be cautious in terms of our expectations when comparing primary tumours and ascites-derived cells, especially when separated by extended periods of time and/or chemotherapy regimens.

**Fig. 5:**
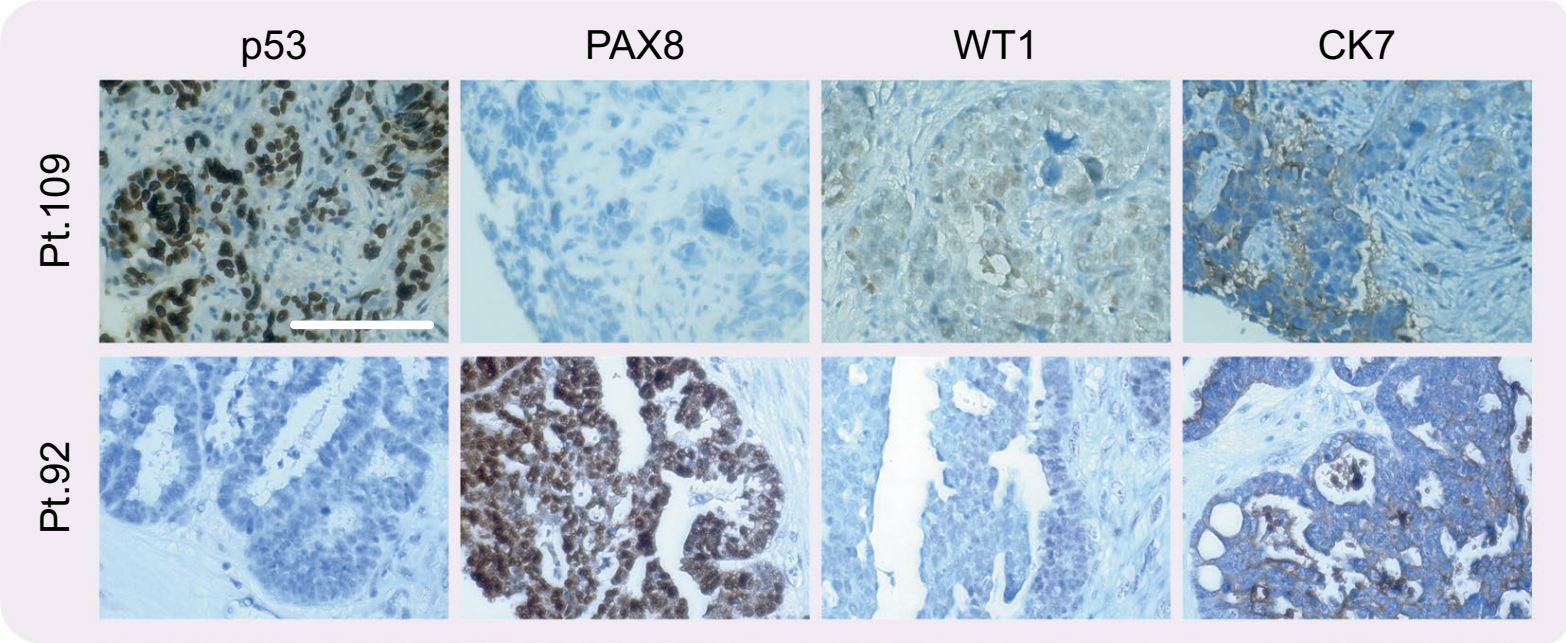
Primary HGSOC can display very different histopathologies. Representative 20× immunohistochemistry images of the primary tumours from patients 92 and 109, stained to detect p53, PAX8, WT1 and Cytokeratin 7. Patient 92 images adapted from Coulson-Gilmer et al. 2021 (License at https://creativecommons.org/licenses/by/4.0/). Scale bar, 100 μm. Panels are representative images from single experiment

Despite the complexity outlined above, we have compared OCMs with their corresponding archival tumour blocks using a panel of standard markers used to diagnose HGSOC in the clinic. Analysis of CK7, PAX8, WT1 and p53 expression (Fig. 5), aided by specialist pathology support, was remarkably congruent (Nelson et al. 2020; Coulson-Gilmer et al. 2021). In addition, targeted amplicon sequencing of primary tumour DNA by a clinically accredited diagnostic service, using a multi-gene panel that included *TP53*, showed excellent congruence with Sanger sequencing of RT-PCR products from matched OCMs.

**Fig. 6:**
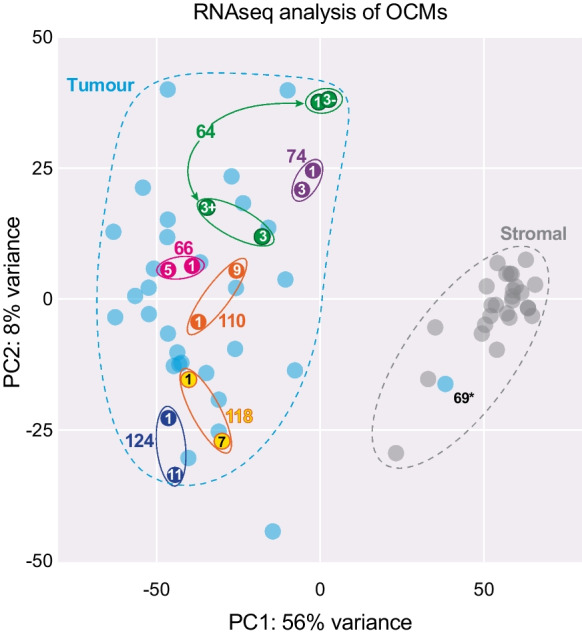
OCM gene expression analysis. Principal component (PC) analysis of RNAseq-derived global gene expression profiles, distinguishing stromal and tumour clades, and showing the close relationship of longitudinal OCMs samples from patients 64, 66, 74, 110, 118 and 124, with numbers inside the symbol indicating the ascites number. 69* is a stromal culture. Published data collated from Nelson et al. 2020, Barnes et al. 2021, Coulson-Gilmer et al. 2021

A related, but perhaps more relevant, question is whether the OCM workflow generates models that reflect the tumour sampled by the respective ascites. Importantly, the pioneering study of Ince et al., demonstrated that the OCMI media maintain the genomic and transcriptomic landscape of the original tumour, and that xenograft tumours show morphology typical of human tumours (Ince et al. 2015). Moreover, by generating OCMs within 5–6 passages, our workflow enables analysis before the expansion of subclonal populations. Additional evidence supporting the generation of reflective models comes from the analysis of OCMs prepared from sequential ascites; in many cases the karyotypes and gene expression profiles are similar (Fig. 6).

**Fig. 7:**
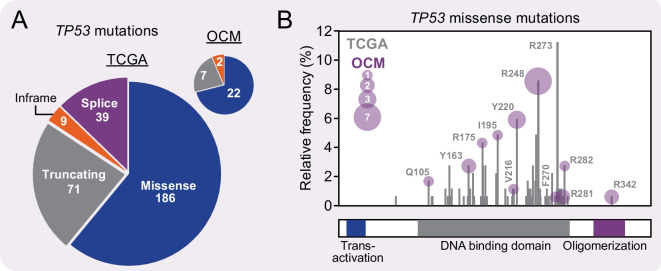
*TP53* mutation profile. **A** Pie charts showing the number of different *TP53* mutation subtypes in the TCGA dataset compared with the subset of OCMs for which *TP53* data is currently available. **B** Comparison of missense *TP53* mutations in the TCGA dataset (grey) versus the OCM subset (purple). OCM data collated from Nelson et al. 2020, Coulson-Gilmer et al. 2021; TCGA data from cBioPortal (Cerami et al. 2012; Gao et al. 2013)

With a take rate of ~30%, another key question is selection bias; does the workflow only select for a subset of HGSOC subtypes? OCM gene expression profiles do display substantial heterogeneity (Fig. 6). In addition, analysis of *TP53* mutations shows that the proportion of missense mutations versus truncating mutations is similar to that described by the TCGA, as is the nature of the missense mutations (Fig. 7) (TCGA 2011; Cerami et al. 2012; Gao et al. 2013). Interestingly however, of the 42 OCMs sequenced so far, we are yet to identify an R273 missense mutation, despite this mutation being the most frequent in the TCGA analysis.

It has been suggested that ex vivo culture may select against *BRCA1/2*-mutant tumours (Hill et al. 2018; Hoffmann et al. 2020; Vias et al. 2023). When we screened a subset of 32 OCMs, 8 were found to be sensitive to PARP inhibition, suggesting an HR-defect (Coulson-Gilmer et al. 2021). Also, in a subset of 20 OCMs derived from patients with known *BRCA1/2* status, seven had germline *BRCA1/2* mutations (Barnes et al. 2021; Coulson-Gilmer et al. 2021). When we analysed OCMs from four of these seven, three harboured *BRCA1/2* mutations (Coulson-Gilmer et al. 2021). Thus, while the number of OCMs fully analysed to date is still relatively small, there is no obvious evidence yet of a selection bias against *BRCA1/2*-mutant or HRD tumours. Indeed, some OCMs appear to reflect the complex mechanisms responsible for drug resistance in patients. Using Rad51 foci formation in response to ionising radiation as a functional readout of HR status, we established that OCM.109 is HRD and harbours a *BRCA1* mutation (Coulson-Gilmer et al. 2021). However, it is PARPi-resistant suggesting a resistance mechanism that bypasses the HR defect. OCM.246 was derived from a patient with a germline *BRCA2* mutation who received olaparib maintenance monotherapy prior to biopsy sampling. Interestingly, this OCM harbours a putative intragenic reversion predicted to restore the *BRCA2* open reading frame, reflecting reversion mechanisms previously described in patients (Christie et al. 2017; Burdett et al. 2023), and the OCM displays intermediate PARPi resistance (Coulson-Gilmer et al. 2021).

Another mechanism of acquired drug resistance in HGSOC is chromosome translocation events leading to upregulation of the drug efflux pump encoded by *ABCB1* (Patch et al. 2015; Christie et al. 2019). A number of OCMs demonstrate upregulated ABCB1 expression and can be re-sensitized to paclitaxel using the efflux inhibitor Elacridar (not shown). Taking all this together, our experience to date is consistent with the notion that the biopsy pipeline and OCM workflow have generated a diverse collection of ovarian cancer models that reflects the disease heterogeneity observed in traditional sample collections. Plus, the biobank reflects various drug resistance mechanisms that have been described previously. As such, the *Living Biobank* provides a unique opportunity to probe aspects of HGSOC biology and explore novel therapeutic strategies.

## OCMs display ongoing CIN

While HGSOC is driven by CIN, mutations in genes directly involved in chromosome replication and segregation are extremely rare in cancer (Matthews et al. 2022). To delineate CIN mechanisms, HGSOC has been studied by whole-genome sequencing (WGS). One landmark study defined two mutational trajectories, the first characterised by HRD, with *BRCA1/2* mutations, amplification of *MYC* and loss of *RB1*; the second characterised by homologous recombination proficiency (HRP) with foldback inversions (FBI) correlating with *CCNE1* amplification and *PTEN* loss (Wang et al. 2017). While elegant, this dualistic model is likely an oversimplification and indeed, a second key study using shallow WGS identified seven CNV signatures, including two HRD signatures and five HRP signatures (Table 1) (Macintyre et al. 2018).

**Table 1:** CNV signatures with associated genomic hallmarks and molecular features. Adapted from (Macintyre et al. 2018)

Signature	Genomic hallmarks	Molecular features
1	Breakage-fusion bridge	Oncogenic RAS-MAPK signalling, e.g. NF1 loss
2	Tandem duplication	CDK12 inactivation
3	HRD type 1	Mutated HR genes (including *BRCA1/2*); PTEN loss
4	Whole-genome doubling	Cell cycle deregulation type 1: MYC, CDK12, Cyclin-E1, PI3K–AKT signalling
5	Chromothripsis	Unknown
6	Focal amplification	Cell cycle deregulation type 2: Cyclin-E1, PI3K–AKT, CCND1, MYC
7	HRD type 2	MYC, Wnt/Interleukin signalling

Matched deep sequencing assigned potential pathways to CNV signatures; one HRD signature was associated with *BRCA1/2* mutations and loss of *PTEN*, while the other was non-*BRCA1/2*-related with *MYC* amplification (Macintyre et al. 2018). HRP signatures were associated with various trajectories including oncogenic RAS, inactivation of CDK12, or cell cycle deregulation (Table 1). Multiple signatures were observed to co-exist in the same sample, including HRD and HRP signatures. Also, the composite signature was predictive, e.g., patients with a high degree of signature 1 had poor prognosis. More recently, a study of 7880 tumours from 33 different tissues was used to devise 17 pan-cancer CNV signatures, including three signatures associated with impaired homologous recombination (IHR) alongside varying degrees of replication stress (Drews et al. 2022). One of these IHR signatures correlated with the two HRD signatures identified in ovarian cancers. Another recent study identified 21 pan-cancer CNV signatures, nine of which were present in ovarian cancer, including one that may be unique to ovarian cancer that could not be assigned to a biological process (Steele et al. 2022).

While ground-breaking, these studies expose important new questions. The presence of multiple signatures is complicated by bulk sequencing archival material; single-cell analyses will be required to disentangle whether individual cells exhibit multiple signatures, or whether this reflects intra-tumour heterogeneity and/or specific microenvironments (Shah 2018). Thus, well-defined in vitro models and/or derived subclones amenable to functional experiments will be required to test hypotheses correlating signatures with cell behaviours (Macintyre et al. 2018).

Our vision is that *Living Biobanks* will provide opportunities to address these issues. Indeed, a key advantage of viable cultures is the ability to analyse highly purified tumour fractions unfettered by contaminating, genetically normal stromal cells, and the microenvironment. Moreover, they are amenable to single-cell analyses, including both shallow WGS and RNA sequencing (Nelson et al. 2020). But most significantly, as viable, proliferating cultures, they can be subjected to functional experiments designed to probe the status of specific signalling and cell cycle pathways, thereby enabling hypotheses that emerge from interrogation of molecular features to be tested more rigorously using phenotypic assays.

As proof-of-principle, to assess CIN functionally in OCMs, we analysed patterns of mitotic chromosome segregation using time-lapse microscopy, facilitated by stable integration of a GFP-tagged histone to visualise the chromatin (Nelson et al. 2020). This revealed highly chaotic and heterogeneous mitoses, with rates of abnormalities far higher than previously observed in established cell lines. Rates of lagging chromosomes, anaphase bridges and cytokinesis/abscission failures were all elevated. The difficulties with chromosome alignment very often resulted in a protracted mitosis, indicating a robust spindle assembly checkpoint. And indeed, when challenged with microtubule toxins, OCMs underwent longer mitotic delays. Interestingly, because of the self-imposed protracted mitosis, we observed several instances of cohesion fatigue (Daum et al. 2011; Stevens et al. 2011); to our knowledge this is the first time this has been seen without experimentally blocking mitosis (Nelson et al. 2020).

While many of the highly abnormal cell divisions did give rise to viable progeny, and certainly sufficient to maintain a proliferative culture, cell fate profiling revealed a number of *dead-ends*, consistent with the notion some genomes are incompatible with life (Nelson et al. 2020). Nevertheless, the extent of mitotic chaos was surprising and suggests that a key feature of HGSOC is deactivation of post-mitotic and/or apoptotic pathways that would normally eliminate genetic deviants. Moreover, it also supports the notion that OCMs provide interesting alternatives to established cell lines for analysing HGSOC CIN mechanisms. Indeed, although established cell lines exhibit ongoing CIN (Lengauer et al. 1997; Penner-Goeke et al. 2017; Tamura et al. 2020), a limited number of subclones tend to dominate (Wangsa et al. 2018), presumably because they represent the fittest, fastest growing cells (Domcke et al. 2013; Ince et al. 2015; Nelson et al. 2020). Accordingly, one might expect that OCMs that start out highly heterogenous would become less complex over time, as the fitter subclones give rise to more progeny with every passage. Empirical evidence supports this. When we analysed spindle poles as a proxy for CIN, comparing OCMs at early and late passages, complexity reduced over time with bipolar spindles becoming more dominant, presumably because they are both already fitter and more likely to give rise to viable daughters (Nelson et al. 2020). This further highlights the advantages of being able to analyse OCMs at early passage when the population is still complex. Ideally, one would want to be able to isolate and expand different subclones; while this is possible (Naffar-Abu Amara et al. 2020), it can be challenging to expand single cells in vitro. However, advances in bar coding technology mean that it is possible to trace lineages without the need for exerting the stress associated with single-cell cloning (Gutierrez et al. 2021). Such bar-coding technologies open up exciting opportunities to study genome evolution and the emergence of drug resistance in patient-derived tumour material.

The heterogenous mitoses described above were observed when the OCMs were cultured as 2D monolayers (Nelson et al. 2020). Interestingly, it has been found that tissue architecture can impact chromosome segregation fidelity (Knouse et al. 2018). In particular, when mouse epithelial cells were cultured as 3D spheroids the rates of chromosome mis-segregation were very low, but this rate increased to ~7% in 2D culture. This raises the possibility that the mitotic errors observed in OCMs may in part be an artefact of in vitro 2D culture. We suspect that this is not the case. In the OCMs, chromosome mis-segregation rates were often around 50%, far higher than was observed in the primary mouse epithelial cells. Moreover, when we specifically grew the OCMs in a 3D environment, we observed equally high rates of segregation error (Nelson et al. 2020). Moreover, we observed additional classes of abnormal mitoses in 3D, including chromosome ejection at anaphase, possibly reflecting the ability of a 3D environment to better anchor ectopic spindle poles.

Taking together the various WGS studies, plus our OCM-derived observations (shallow scWGS karyotyping, time-lapse microscopy, and more traditional M-FISH-based karyotyping) (Nelson et al. 2020), a very consistent picture emerges — HGSOC genomes are highly dynamic, undergoing persistent and high rates of CIN. A key next step will be to align these different modalities and integrate CIN signatures with mutational profiles derived from bulk WGS data and gene expression signatures from RNA sequencing. This multi-omics data can then be aligned with clinical outcome data, as well as functional phenotypes derived from cell-based analysis and drug-sensitivity profiling to test hypotheses (see below). Thus, the OCMs represent an invaluable resource to delineate mechanisms underlying aberrant mitoses and CIN in HGSOC cells. Of particular value will be matched longitudinal OCMs, especially those that include OCMs from both chemotherapy-naïve and post-treatment disease, to better understand how CIN drives the emergence of drug resistance in patients.

## A platform for drug discovery

CIN has the ability to drive the emergence of drug resistance in patients; for example, chromosome translocations within *ABCB1* can lead to overexpression of the MDR1/p-glycoprotein drug efflux pump (Patch et al. 2015; Christie et al. 2019). Importantly, we identified *ABCB1* translocations in a number of OCMs and have shown that drug sensitivity can be restored by co-exposure with efflux inhibitors (not shown). Interestingly, in OCM.246 we identified three different *ABCB1* translocations (Williams et al. 2020), as well as a putative *BRCA2* reversion mutation (Coulson-Gilmer et al. 2021), illustrating both the incredible capacity of CIN to alter the genome and the intense selective pressure that chemotherapy exerts. Also, these observations provide further evidence that OCMs provide a window into the drug resistance mechanisms seen in patients (Patch et al. 2015; Christie et al. 2017; Christie et al. 2019; Burdett et al. 2023), and that OCMs provide a potentially interesting platform for drug-sensitivity profiling to complement multi-omics analyses.

To measure drug sensitivities of OCMs, we have optimised a high-throughput assay that uses object counting to measure proliferation (Nelson et al. 2020; Coulson-Gilmer et al. 2021; Golder et al. 2022). In brief, OCMs expressing a GFP-tagged histone are analysed by time-lapse microscopy and changes in green object count over time are used as a proxy for proliferation. The doubling time is then calculated by determining the inverse gradient of the linear portion of a log_2_ transformation of the fluorescent object count, normalised to *t* = 0 h (Golder et al. 2022). This approach has advantages over traditional end-point viability assays that infer cell viability by measuring ATP metabolism, which can be confounded by cytostatic effects whereby cells stop proliferating but remain metabolically active (Niepel et al. 2019). Moreover, the approach is very data rich, providing single-cell-level resolution over time. Interrogating time-lapse sequences can provide additional information in terms of cell fate and behaviour simply not apparent in population-based end-point assays. Using this approach, we have measured proliferation rates of numerous OCMs (Pillay et al. 2019; Nelson et al. 2020; Coulson-Gilmer et al. 2021; Golder et al. 2022).

Analysing proliferation in response to drug exposure then enables drug-sensitivity profiling. In brief, we determine the half maximal growth inhibition concentration of drug (GI_50_) using dose-response curves generated by measuring the area-under-the-curve of fluorescent object count over time for a range of drug concentrations (Golder et al. 2022). The high-throughput nature enables multiple technical replicates and the tractability of the OCMs enables biological replicates to be analysed in quick succession. Various parameters can influence multi-well assay readouts, and recently we explored a number of parameters including cell seeding density and assay duration, as well as analytical approaches to account for variability in cell cycle duration (Golder et al. 2022). While there is heterogeneity due to the complex nature of the OCMs, estimates of doubling times were largely consistent when remeasured 18 months apart, and ex vivo responses to platinum largely reflected patient responses (Nelson et al. 2020; Golder et al. 2022). Thus far, we have evaluated OCM sensitivity to cisplatin, paclitaxel and inhibitors targeting PARP-1/2 and the PAR glycohydrolase (Pillay et al. 2019; Nelson et al. 2020; Coulson-Gilmer et al. 2021). More recently, screening a panel of 16 diverse OCMs, using all possible one-, two-, three- and four-drug combinations of four inhibitors targeting the DNA replication stress response at GI_10_ concentrations (240 assays in total), demonstrated that the low-dose combination of ATR and CHK1 inhibitors had significant activity against 15 OCMs, identifying a potentially novel therapeutic strategy (Golder et al. 2022).

Due to the extensive proliferative potential of OCMs, drug sensitivity can also be assessed by longer-term colony formation assays, thereby complementing the shorter-term time-lapse-based proliferation assays (Coulson-Gilmer et al. 2021). While OCMs can be cultured in a 3D context, thus far we have measured drug sensitivity of 2D monolayers. Because the microenvironment can influence drug sensitivity, it will be interesting to analyse in vitro chemotherapy responses in more complex 3D and co-culture models (Tomas and Shepherd 2023). However, it is noteworthy that sensitivity and resistance to PARPi, which arguably represent the most significant advancement in recent years for treating patients with HGSOC, manifests very clearly when analysing OCMs as 2D monolayers (Coulson-Gilmer et al. 2021). Indeed, our focus is on exploiting cell cycle vulnerabilities intrinsic to the tumour cells, vulnerabilities that may be less sensitive to the tumour microenvironment. Furthermore, the impact of the microenvironment on response to therapy is highly complex, for example extracellular matrix components have been associated with both chemotherapy sensitivity and resistance (Ahmed et al. 2007; Etemadmoghadam et al. 2009; Helleman et al. 2010; Kozlova et al. 2020; Guo et al. 2021). Clearly, these interactions bring about additional complexity, therefore our approach in the first instance is to focus on dissecting intrinsic tumour cell properties and drug responses. Armed with this knowledge, we will be better placed to explore how the microenvironment modulates tumour cell biology. Importantly, a number of OCMs have been successfully engrafted in immunocompromised mice to form xenograft tumours. These OCM-derived xenograft (ODX) models retain the molecular features of the original OCM, both in vivo and ex vivo following excision and disaggregation (not shown). Such ODX models will provide excellent opportunities to test new therapeutic strategies in vivo that emerge from drug sensitivity profiling of 2D monolayers.

## Future perspectives

The *Living Biobank* currently contains over 120 OCMs from more than 80 patients and is expanding at a rate of 2–3 new OCMs per month. Fifteen ascites-derived OCMs are chemo-naïve and, as the biobank grows, the number of longitudinal cohorts with matched post-treatment OCMs will expand. While focused on HGSOC, we are also collecting smaller cohorts of OCMs derived from other ovarian cancer subtypes as these provide interesting comparators. Although ascites offer various advantages, we have recently received more solid samples (and currently have eight solid-derived OCMs), which increases the potential to broaden the diversity of the biobank. Thus, in summary, the pipeline and workflow we have developed has allowed assembly of a large and diverse collection of ovarian cancer models that reflect the diversity of HGSOC. Importantly, the experimental tractability of OCMs in terms of integrating multi-omics data, including single cell approaches, with functional assays, including high-resolution cell biology approaches and drug-sensitivity profiling, opens up new opportunities to delineate the molecular mechanisms responsible for driving CIN in this particular disease. An important future goal is also to collate the wealth of data associated with the OCMs in a searchable format, so that it is available to researchers in the ovarian cancer community alongside the OCMs.

## Data Availability

Datasets supporting this review article are published previously (Nelson et al. 2020; Barnes et al. 2021; Coulson-Gilmer et al. 2021; Golder et al. 2022). Further information and reagent requests may be directed to Stephen S. Taylor (stephen.taylor@manchester.ac.uk).

## Abbreviations

CIN: Chromosome instability
CN: Chemotherapy-naïve
CNV: Copy number variation
CRC: Colorectal cancer
HGSOC: High-grade serous ovarian cancer
HR: Homologous recombination
HRD: Homologous recombination deficient
HRP: Homologous recombination proficiency
LGSOC: Low-grade serous ovarian cancer
IHR: Impaired homologous recombination
OCM: Ovarian cancer model
OCMI: Ovarian carcinoma modified Ince
ODX: OCM-derived xenograft
PARPi: PARP-1/2 inhibitor
PC: Principal component
RNAseq: RNA sequencing
scWGS: Single-cell whole-genome sequencing
WGS: Whole-genome sequencing
